# Updated Surveillance Metrics and History of the COVID-19 Pandemic (2020-2023) in Latin America and the Caribbean: Longitudinal Trend Analysis

**DOI:** 10.2196/44398

**Published:** 2024-05-17

**Authors:** Lori Ann Post, Scott A Wu, Alan G Soetikno, Egon A Ozer, Yingxuan Liu, Sarah B Welch, Claudia Hawkins, Charles B Moss, Robert L Murphy, Maryann Mason, Robert J Havey, Alexander L Lundberg

**Affiliations:** 1 Buehler Center for Health Policy and Economics Robert J Havey, MD Institute for Global Health Northwestern University Chicago, IL United States; 2 Department of Emergency Medicine Feinberg School of Medicine Northwestern University Chicago, IL United States; 3 Feinberg School of Medicine Northwestern University Chicago, IL United States; 4 Department of Medicine Division of Infectious Diseases, Feinberg School of Medicine Northwestern University Chicago, IL United States; 5 Center for Pathogen Genomics and Microbial Evolution Robert J Havey, MD Institute for Global Health Northwestern University Chicago, IL United States; 6 Center for Global Communicable and Emerging Infectious Diseases Robert J Havey, MD Institute for Global Health Northwestern University Chicago, IL United States; 7 Institute of Food and Agricultural Sciences University of Florida Gainesville, FL United States; 8 Robert J Havey, MD Institute for Global Health Northwestern University Chicago, IL United States; 9 Department of Medicine, General Internal Medicine and Geriatrics Feinberg School of Medicine Northwestern University Chicago, IL United States

**Keywords:** SARS-CoV-2, COVID-19, Latin America, Caribbean, pandemic, surveillance, COVID-19 transmission, speed, acceleration, deceleration, jerk, dynamic panel, generalized method of moments, GMM, Arellano-Bond, 7-day lag, epidemiological, pandemic, genomic, transmission

## Abstract

**Background:**

In May 2020, the World Health Organization (WHO) declared Latin America and the Caribbean (LAC) the epicenter of the COVID-19 pandemic, with over 40% of worldwide COVID-19–related deaths at the time. This high disease burden was a result of the unique circumstances in LAC.

**Objective:**

This study aimed to (1) measure whether the pandemic was expanding or contracting in LAC when the WHO declared the end of COVID-19 as a public health emergency of international concern on May 5, 2023; (2) use dynamic and genomic surveillance methods to describe the history of the pandemic in the region and situate the window of the WHO declaration within the broader history; and (3) provide, with a focus on prevention policies, a historical context for the course of the pandemic in the region.

**Methods:**

In addition to updates of traditional surveillance data and dynamic panel estimates from the original study, we used data on sequenced SARS-CoV-2 variants from the Global Initiative on Sharing All Influenza Data (GISAID) to identify the appearance and duration of variants of concern (VOCs). We used Nextclade nomenclature to collect clade designations from sequences and Pangolin nomenclature for lineage designations of SARS-CoV-2. Additionally, we conducted a 1-sided *t* test for whether the regional weekly speed (rate of novel COVID-19 transmission) was greater than an outbreak threshold of 10. We ran the test iteratively with 6 months of data across the period from August 2020 to May 2023.

**Results:**

The speed of pandemic spread for the region had remained below the outbreak threshold for 6 months by the time of the WHO declaration. Acceleration and jerk were also low and stable. Although the 1- and 7-day persistence coefficients remained statistically significant for the 120-day period ending on the week of May 5, 2023, the coefficients were relatively modest in magnitude (0.457 and 0.491, respectively). Furthermore, the shift parameters for either of the 2 most recent weeks around May 5, 2023, did not indicate any change in this clustering effect of cases on future cases. From December 2021 onward, Omicron was the predominant VOC in sequenced viral samples. The rolling *t* test of speed=10 became entirely insignificant from January 2023 onward.

**Conclusions:**

Although COVID-19 continues to circulate in LAC, surveillance data suggest COVID-19 is endemic in the region and no longer reaches the threshold of the pandemic definition. However, the region experienced a high COVID-19 burden in the early stages of the pandemic, and prevention policies should be an immediate focus in future pandemics. Ahead of vaccination development, these policies can include widespread testing of individuals and an epidemiological task force with a contact-tracing system.

## Introduction

### Background

COVID-19, the disease caused by the virus SARS-CoV-2, was first detected in Wuhan, China, in the fall of 2019 [1-5]. The first case of COVID-19 in Latin America and the Caribbean (LAC) [6] occurred in São Paulo, Brazil, and involved a Brazilian national traveling home from Northern Italy [7]. Our research team conducted an analysis of the pandemic in LAC 1 year into the pandemic [8]. This study provides 2 additional years of updated surveillance and epidemiological analysis for the region.

We have adopted the World Bank’s definition of LAC, which is based on economic development and geographical proximity, encompassing Argentina, Belize, Bolivia, Brazil, Colombia, Chile, Costa Rica, Ecuador, El Salvador, Guatemala, Guyana, Honduras, Mexico, Nicaragua, Panama, Paraguay, Peru, Suriname, Uruguay, and Venezuela in the Latin America region and Antigua and Barbuda, Aruba, the Bahamas, Barbados, Bermuda, the British Virgin Islands, the Cayman Islands, Cuba, Curaçao, Dominica, the Dominican Republic, Grenada, Haiti, Jamaica, Puerto Rico (the United States), St Barthelemy, St Kitts and Nevis, St Lucia, St Vincent and the Grenadines, Sint Maarten (the Netherlands), Trinidad and Tobago, Turks and Caicos Islands, and the US Virgin Islands in the Caribbean region [6].

The World Health Organization (WHO) and Director-General Ghebreyesus declared the end of COVID-19 as a public health emergency of international concern on May 5, 2023 [9-11], based on the recommendation of the COVID-19 Emergency Committee [11]. To that end, we compare how the pandemic was progressing before and after the declaration. The “public health emergency of international concern” status is not a definitive statement of whether a pandemic exists nor whether one has ended. The aim of this study was to provide a statistical context to determine whether COVID-19 had transitioned from pandemic to endemic status in LAC at the point of the declaration, a time when many governments worldwide had dropped emergency policy responses around COVID-19.

Epidemiological terms, such as “pandemic,” “epidemic,” “outbreak,” and “endemic,” are used to describe the occurrence and spread of diseases [2,12]. The distinctions between these terms lie in their scope and geographic extent. The terms “pandemic,” “epidemic,” “outbreak,” and “endemic” do not describe the severity of a disease; instead, they describe its prevalence [13]. An epidemic refers to a sudden increase in the number of disease cases in a specific population or region. If the epidemic spreads across several countries or continents, it becomes a pandemic. An outbreak, in contrast, describes a sudden increase in a concentrated setting, usually involving a more limited geographic area than an epidemic. The word “endemic” refers to the constant presence of a disease in a particular geographic region or population, with no sudden increases in case volume [14-16].

Public health surveillance is the “ongoing, systematic collection, analysis, and interpretation of health-related data essential to planning and evaluation of public health practice” [17]. Surveillance not only explains the burden due to a disease but also generates research questions and guides researchers on topics that require further investigation [18-32]. Surveillance allows us to compare the burden of a disease between geographical regions and to understand which regions are most impacted. The impact can be measured through absolute numbers and rates of how many people contract a disease, how many die, and what the affiliated costs are.

However, traditional public health surveillance has inherent limitations, which this study addresses. For example, surveillance predominantly offers a retrospective view, presenting a static snapshot of past events [18-32]. In the middle of a burgeoning pandemic, policy makers and public health practitioners also need to understand what is about to happen. For example, forward-looking metrics are helpful to understand the trajectory of an outbreak’s growth, the shift from linear to exponential expansion, or disparities in the impact across regions. To that end, we have developed enhanced surveillance metrics that capture the dynamic nature of a pandemic and identify imminent growth, notably pinpointing a region’s position along the epidemiological outbreak curve. Although many large cities, states, and countries conducted helpful COVID-19 disease projection models to inform leaders how to “flatten the curve” so as not to overwhelm health systems, these one-off studies cost millions and only gave projections for a relatively short time frame [33-49]. Moreover, these studies were outdated by the time of their publication and were not relevant to future variants of concern (VOCs) with varied rates of transmission, severe disease, and reproduction. Our enhanced surveillance metrics provide predictive models in an ongoing systematic fashion consistent with surveillance systems. Our enhanced metrics also incorporate dynamic indicators of the pandemic speed at national, regional, and worldwide levels. These metrics gauge the acceleration of the speed from 1 week to the next and predict new cases based on prior dynamic panels across months of daily infections, effectively forecasting the progression of the outbreak. This predictive ability identifies the “echoing forward” of cases. Our metrics have undergone rigorous testing and validation in prior publications that this study updates [8,50-57].

For purposes of this study, standard surveillance metrics explain what has already happened in LAC, while enhanced surveillance metrics show what is about to happen by predicting new cases based on past infections or where along an epidemiological curve a country may sit. We use both types of metrics to inform the possible declaration of the end of the pandemic.

### Objectives

This study had 3 objectives. First, we aimed to measure whether there was an expansion or contraction in the pandemic in LAC when the WHO declared the end of the COVID-19 public health emergency of international concern on May 5, 2023. At both the region and country level, we used advanced surveillance and analytical techniques to describe the status of the pandemic in a 2-week window around the WHO declaration. From a public health perspective, we needed to know whether the rate of new COVID-19 cases was increasing, decreasing, or stable from week to week and whether any changes in the transmission rate indicated an acceleration or deceleration of the pandemic. Statistical insignificance represents a significant finding, as this can signal the epidemiological “end” to the pandemic if the rate of new cases is 0 (or extremely low) and stable, meaning that the number of new cases is neither accelerating nor decelerating.

Second, we used dynamic and genomic surveillance methods to describe the history of the pandemic in the region and situate the time window around the WHO declaration within the broader history. We included the ratio of COVID-19 deaths to the number of transmissions as a proxy for the mortality risk from infection at the population level. We also included a historical record of genomic surveillance from sequenced viral specimens to identify the appearance and spread of VOCs in the region.

Third, we aimed to provide a historical context for the course of the pandemic in LAC. We addressed several questions: How did countries respond to the pandemic? How did the region fare in terms of the disease burden? What social, economic, and political factors shaped the course of COVID-19 in the region? This context can provide important lessons for disease prevention and mitigation in future pandemics.

Regional analyses are valuable because multiple factors, not limited to climate change, governance, economic trade routes, public health infrastructure, and human migration, can all shape the risk profile of COVID-19 transmissions [58-62]. In this study, we discussed the role of these factors in the course of the pandemic in LAC. In May 2020, the WHO declared LAC the epicenter of the pandemic, accounting for over 40% of all worldwide COVID-19 deaths at the time [63]. This high disease burden was a result of the unique circumstances in LAC, and these circumstances can inform preparedness for future pandemics and waves of transmissions.

## Methods

### Study Design and Data Collection

We conducted trend analyses with longitudinal COVID-19 data from Our World in Data (OWID) [64] to provide updates of traditional surveillance data and dynamic panel estimates from the original studies by Post and colleagues [8,55,56,65-67]. For the LAC region, the data comprised an unbalanced panel of 43 countries and territories, from August 21, 2020, to May 12, 2023. Please see the *Introduction* section for the complete list of countries. Since several countries worldwide switched from daily to weekly reports at various points in 2023, so we used a cubic spline to interpolate daily new cases and deaths if any country had 4 consecutive periods of nonzero new cases interspersed by 6 days of 0 new cases.

Additionally, we used data on sequenced SARS-CoV-2 variants from the Global Initiative on Sharing All Influenza Data (GISAID) to identify the appearance and duration of VOCs [68-72]. We used Nextclade nomenclature [73] to collect clade designations from sequences and Pangolin nomenclature for lineage designations of SARS-CoV-2 [74,75]. Metadata for the study period were collected on June 22, 2023. To avoid a low frequency or potentially erroneous samples, the data set was further filtered to exclude months with fewer than 100 available samples, variant groups with fewer than 5 samples in a month, and variant groups representing less than 0.5% of the total samples in a month. The final data set consisted of 184,386 total samples available on GISAID [69-72].

Traditional surveillance metrics include the speed of spread of the pandemic, defined as the rate of new COVID-19 cases per 100,000 people. Enhanced metrics add acceleration, jerk, and 1- and 7-day persistence measures. Acceleration is the change in speed from one unit of time to the next. This measure can identify whether the rate of transmission is increasing (positive acceleration), decreasing (negative), or stable (0). Jerk is the change in acceleration from one time unit to the next. Its name is lifted from physics nomenclature. A large jerk can signal explosive growth in transmission rates. The 1- and 7-day persistence measures provide the impact of the 1- and 7-day lag of speed on the current speed. Thus, these measures capture how COVID-19 cases echo-forward to cases either 1 or 7 days later. They are derived from an Arellano-Bond dynamic panel data model [76], which follows the form

y_it_ = ρy_it – 1_ + βX_it_ + α_i_ + u_it_,

where the dependent variable is speed, the independent variables include weekend and recent week indicators, α_i_ is a country fixed effect, and u_it_ is the idiosyncratic error term. See the initial study for details [8].

### Statistical Analysis

Lastly, we analyzed the potential “statistical end” to the pandemic with (1) dynamic panel estimates for shifts in the pandemic [40,42] and (2) a 1-sided *t* test for whether the mean speed was equal to or greater than the outbreak threshold of 10 [77]. We ran the latter test on a rolling 6-month window of the weekly regional speed, and we plotted the *P* values from the *t* test over time. All statistical analyses were conducted in R version 4.2.1 (R Foundation for Statistical Computing) with the *plm* package (version 2.6-2) [65,66].

### Ethical Considerations

All data used in this study are publicly available and contain no identifiable, private information. Therefore, the study does not constitute research with human subjects, as defined by 45CFR46:102, and Institutional Review Board review was unsolicited. However, the authors note that anonymized COVID-19 data surveillance systems can generate local and global ethical questions beyond the scope of this study [78].

## Results

### Dynamic Panel Estimates

Table 1 presents dynamic panel estimates for the most recent time window. The Wald test for regression was significant (*P*<.001), and the Sargan test failed to reject the validity of the overidentification restrictions (*P*=.99). Although the 1- and 7-day lag coefficients were statistically significant as a function of the large sample size, suggesting a cluster effect in which cases on a given day impacted cases 1 and 7 days later, the coefficients were modest in magnitude (0.457 and 0.491, respectively). Furthermore, the shift parameters for the most recent week were significant and negative, meaning the clustering effect had become smaller in the week after the WHO declaration (but the shift parameter was positive and similar in magnitude for the prior week).

**Table 1:** Arellano-Bond dynamic panel estimates of the number of daily COVID-19 infections reported by LAC^a^ countries from April 28 to May 12, 2023.^b^

Variable	Value	*P* value
1-day lag coefficient	0.457	<.001
7-day lag coefficient	0.491	<.001
Shift parameter week of April 28	0.212	.03
Shift parameter week of May 5	–0.274	<.001
Weekend	0.981	.08

^a^LAC: Latin America and the Caribbean.

^b^Wald test: *χ*^2^_6_=951.65 (*P*≤2.22e-16); Sargan test: *χ*^2^_540_=37 (*P*=.99).

### Static Surveillance Metrics

Static surveillance metrics for the week of April 28, 2023, are provided in Table 2. The same metrics for the week of May 5, 2023, are provided in Table S1 in Multimedia Appendix 1. Most countries had a small number of new COVID-19 cases. The exceptions were Brazil, Costa Rica, Panama, Puerto Rico, and Uruguay. By far, the highest transmission rate was observed in Puerto Rico, where the speed was 144 in the week of April 28 and 182 in the following week. The next highest speed over the 2 weeks was just under 19 in Brazil, and the speed for the country dipped to 12 in the following week. For all other countries and territories, the speed was below the threshold considered a low transmission rate by the Centers for Disease Control and Prevention (CDC) [79]. Most rates fell well below the informal threshold of 10 cases per week per 100,000 population [8,50-57]. Specifically, *low* transmission is considered no more than 10 cases per 100,000 people per week, *moderate* transmission is 10-50 cases per 100,000 people per week, and *substantial* transmission is 50-100 cases per 100,000 people per week [79,80].

**Table 2:** Static COVID-19 surveillance metrics for LAC^a^ countries in the week of April 28, 2023.

Country	New COVID-19 cases, n	Cumulative COVID-19 cases, n	7-day Moving average of new cases	Weekly transmission rate/100,000 individuals	New deaths, n	Cumulative deaths, n	7-day Moving average of deaths	Death rate/100,000 individuals	Conditional death rate
Argentina	0	10,044,957	0	0	0	130,472	0	0	0.01
Aruba	10	44,114	10	0	0	287	0	0	0.01
Barbados	0	107,466	0	0	0	588	0	0	0.01
Belize	0	70,782	0	0	0	688	0	0	0.01
Bermuda	0	18,860	0	0	0	165	0	0	0.01
Bolivia	61	1,197,239	60.57	3.48	0	22,377	0.14	0.02	0.02
Brazil	5827	37,449,418	5953.43	18.95	0	701,494	39.86	0.15	0.02
Chile	208	5,283,908	273	7.44	0	61,384	8.71	0.28	0.01
Colombia	89	6,364,636	89.29	1.20	0	142,713	1	0.02	0.02
Costa Rica	139	1,228,659	151.29	18.81	0	9351	1.71	0.23	0.01
Cuba	44	1,113,088	37.29	0	0	8530	0	0	0.01
Curacao	2	45,798	1.71	6.69	0	301	0	0.56	0.01
Dominican Republic	7	661,045	5.71	0	0	4384	0	0	0.01
Ecuador	126	1,061,766	108	4.89	0	36,019	0	0	0.03
Grenada	0	19,693	0	0	0	238	0	0	0.01
Guatemala	85	1,248,171	97.14	0	0	20,189	0	0	0.02
Guyana	1	73,162	3	0	0	1298	0	0	0.02
Haiti	0	34,228	0.29	0	0	860	0	0	0.03
Honduras	0	472,533	0	0	0	11,112	0	0	0.02
Jamaica	9	154,786	7.43	2.24	0	3536	0	0.28	0.02
Mexico	1182	7,590,885	1264.14	6.49	0	333,990	9.43	0.04	0.04
Nicaragua	1	15,697	1	0.10	0	245	0	0	0.02
Panama	86	1,036,733	89	13.67	0	8621	0.43	0.08	0.01
Paraguay	0	735,759	0	0	0	19,880	0	0	0.03
Peru	280	4,501,130	262.29	5.77	0	220,122	7	0.10	0.05
Puerto Rico	670	1,224,366	606.71	144.29	0	5901	1.57	0.33	0
St Barthelemy	1	5486	0.14	0	0	5	0	0	0
Sint Maarten	0	11,030	0	0	0	92	0	0	0.01
St Kitts and Nevis	0	6598	0	0	0	46	0	0	0.01
St Lucia	0	30,052	0	0	0	409	0	0	0.01
St Vincent and Grenadines	1	9611	1	0	0	124	0	0	0.01
Suriname	0	82,495	0	0	0	1404	0	1.22	0.02
Trinidad and Tobago	7	191,350	–1.57	3.01	0	4387	0	–0.21	0.02
Turks and Caicos Islands	4	6565	4.57	0	0	38	0	0	0.01
US Virgin Islands	2	24,919	2	0	0	130	0	0	0.01
Uruguay	87	1,037,893	92.43	17.86	0	7625	0	0.16	0.01
Venezuela	7	552,578	3.57	0	0	5856	0	0	0.01

^a^LAC: Latin America and the Caribbean.

We noted, however, some sparsity in data reports. To temper the episodic reporting that became more frequent in 2023, we shifted from daily transmission rates and daily speed to weekly transmission rates and weekly speed in contrast to our initial study. A second benefit of shifting from daily to weekly reporting was that the CDC established weekly thresholds of what constitutes a low, moderate, and substantial outbreak, as noted earlier. For example, having 0 new cases in Argentina over the 2 weeks is clearly a product of a failure to report data. Although Puerto Rico appears to have been in a large outbreak around the WHO declaration, island territories often vacillate between high and low transmission rates [81]. The outbreaks in the other 4 countries were relatively small. Based on the definition of a pandemic or an outbreak in several countries, the data indicated a shift from pandemic to endemic COVID-19 in most of LAC, while it was an epidemic in Puerto Rico.

The surveillance metrics demonstrated little to no change before and after the WHO declaration. Without question, Brazil had the most cases of COVID-19 transmissions and deaths, but this rank is partly a function of population size. Thus, a better measure is the number of COVID-19 cases and deaths per 100,000 people. Moreover, death is often a better proxy for the state of an outbreak than transmissions because deaths are less likely to be undercounted [82]. Undercounting may be due to poor public health infrastructure, a switch to home antigen testing, or a dearth of polymerase chain reaction (PCR) testing or other resources. Although Brazil reported 0.02 deaths per confirmed infection, several countries had higher rates. Peru had the highest rate at 0.05, followed by Mexico at 0.04 and Ecuador, Haiti, and Paraguay at 0.03.

Table 3 and Table S2 in Multimedia Appendix 1 contain enhanced dynamic surveillance metrics for the 2 weeks before and after May 5, 2023. Again, the speed was low for every country except Brazil, Costa Rica, Panama, Puerto Rico, and Uruguay. Acceleration and jerk were both either small or negative for every country and territory. The 7-day persistence effect on the speed was also small except for the countries in outbreak status. These metrics suggest the pandemic may have indeed ended for the region. Only a single territory was in a substantial outbreak and 4 others in relatively modest outbreaks; thus, epidemiologically, COVID-19 would be considered an epidemic in Puerto Rico and an end-stage epidemic in Brazil, Costa Rico, Panama, and Uruguay, with mild outbreaks in these countries. The average speed for the LAC region was below the outbreak threshold. Since COVID-19 cases in LAC have leveled off, with the exception of Puerto Rico, indicating that the disease is stable or constant in the region, it is more accurate to say that COVID-19 in LAC has shifted to being endemic [13,83,84]. Note that the numbers in Table 3 and Table S2 in Multimedia Appendix 1 are not calculated as day-over-day averages across the week, as they are in Table 2 and Table S1 in Multimedia Appendix 1. Thus, the magnitudes of the speed are consistent but not identical to those matched across tables.

Table 4 compares the 7-day persistence effect on the speed for the top 5 countries around the 2 weeks of the WHO declaration. These ranks largely reflect the speed in the countries with outbreaks in the prior tables. However, Chile appeared at rank 5 in the week of April 28, 2023. This level of persistence would be cause for alarm if Chile did not see a substantial drop, as it did, in the subsequent week.

**Table 3:** Novel COVID-19 surveillance metrics for LAC^a^ countries for the week of April 28, 2023.

Country	Weekly speed	Weekly acceleration	Weekly jerk	7-day Persistence effect on speed
Argentina	0	0	0	0
Aruba	0	0	0	0
Barbados	0	0	0	5.23
Belize	0	0	0	0
Bermuda	0	0	0	0
Bolivia	3.47	0.02	–0.01	2.55
Brazil	19.35	–0.24	0.07	17
Chile	9.74	–0.80	0.02	12.09
Colombia	1.20	0.01	–0.01	0.77
Costa Rica	20.44	–0.43	–0.07	17.59
Cuba	0	0	0	0.10
Curacao	5.90	0.24	0.02	3.47
Dominican Republic	0.05	0	0	0.05
Ecuador	4.20	0.23	0	2.42
Grenada	0	0	0	0.88
Guatemala	0.60	0	0	0.43
Guyana	0.51	0	0	0.16
Haiti	0	0	0	0.01
Honduras	0	0	0	0.05
Jamaica	1.86	0.10	0.02	1.32
Mexico	6.94	–0.12	–0.03	5.60
Nicaragua	0.09	0	0	0.04
Panama	14.12	–0.05	–0.04	11.86
Paraguay	0	0	0	0
Peru	5.40	0.05	0.06	4.94
Puerto Rico	130.59	4.03	0.40	85.52
St Barthelemy	1.30	0	0	1
Sint Maarten	0	0	0	0
St Kitts and Nevis	0	0	0	0
St Lucia	0	0	0	0
St Vincent and Grenadines	0	0	0	0.95
Suriname	0	0	0	0
Trinidad and Tobago	–0.76	0.60	0.47	2.29
Turks and Caicos Islands	0	0	0	0
US Virgin Islands	1.72	0	0	1
Uruguay	18.94	–0.40	0.03	17.12
Venezuela	0.01	0	0	0.05
Argentina	0	0	0	0

^a^LAC: Latin America and the Caribbean.

**Table 4:** LAC^a^ countries with the highest 7-day persistence estimate in the weeks of April 28 and May 5, 2023.

Week of April 28, 2023		Week of May 5, 2023		
Country	7-day Persistence		Country	7-day Persistence
Puerto Rico	85.52		Puerto Rico	91.89
Costa Rica	17.59		Costa Rica	14.38
Uruguay	17.12		Brazil	13.62
Brazil	17.00		Uruguay	13.33
Chile	12.09		Panama	9.94

^a^LAC: Latin America and the Caribbean.

**Figure 1:**
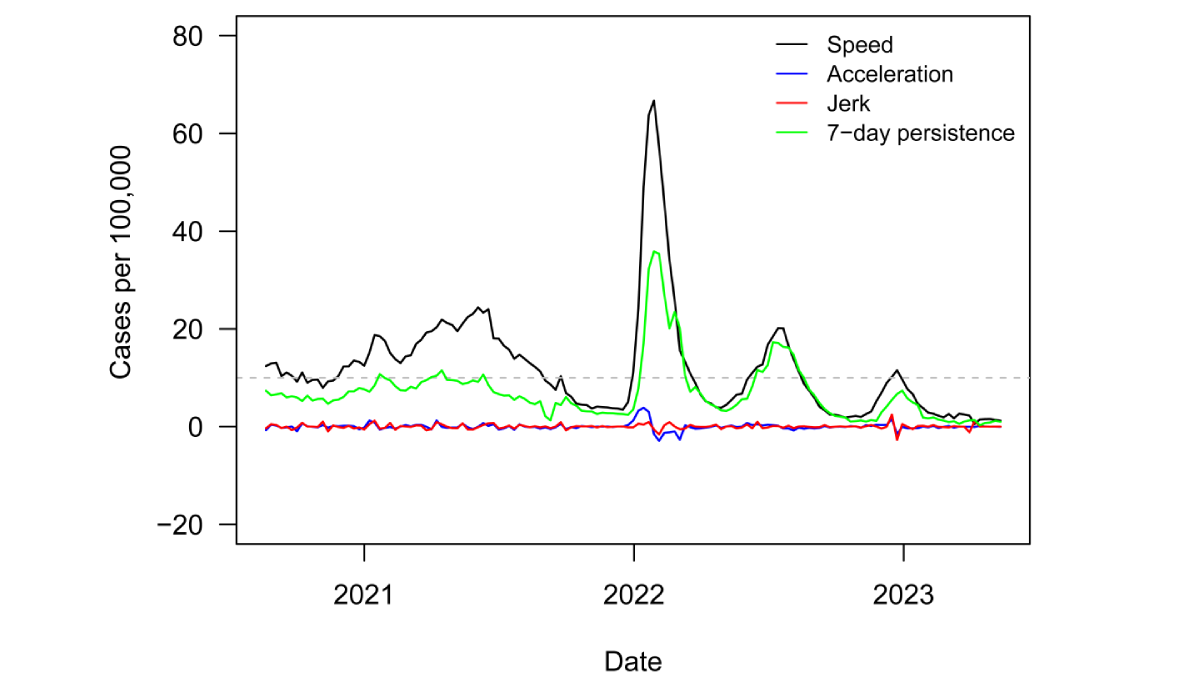
Timeline of speed, acceleration, jerk, and 7-day persistence in LAC from 2020-2023. LAC: Latin America and the Caribbean.

Figure 1 plots the regional speed, acceleration, jerk, and 7-day persistence metrics from August 21, 2020, to May 12, 2023. The dashed gray line denotes the informal CDC outbreak threshold speed of 10. The region was in a nearly continuous state of outbreak until September 2021. Several months later, the Omicron variant drove a substantial outbreak, culminating in a speed of 66 novel cases per week per 100,000 population. A second, smaller wave followed, which brought a peak speed of 20 in July 2022. A final outbreak barely eclipsed the outbreak threshold speed of 10 in December 2023. The region has seen the speed decrease, stabilize, and remain well below the outbreak threshold ever since.

**Figure 2:**
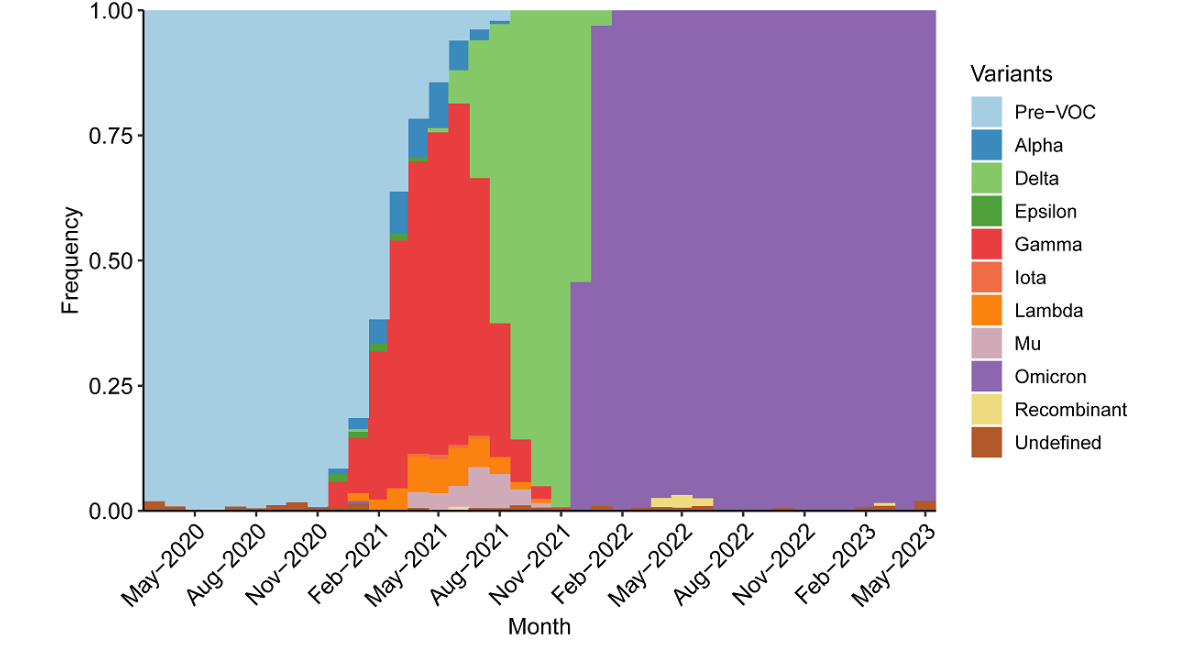
VOC groups as a proportion of all sequenced SARS-CoV-2 specimens over time in LAC from March 2020 to May 2023. LAC: Latin America and the Caribbean; VOC: variant of concern.

Figure 2 plots variant groups as a proportion of all viral specimens collected and sequenced in the region (and made available through GISAID) each month. The early, nearly continuous state of outbreak in the region spanned the ancestral variant through the temporary dominance of the Delta variant. Interestingly, the region also had a period in which the majority of viral specimens returned as the Gamma variant. Compared to other worldwide regions, LAC had a greater diversity of variants. Later outbreaks were driven by the Omicron variant. LAC, like much of the rest of the world, saw a surge in cases amid the heightened transmissibility of Omicron [85]. Another potential indication of the end to the pandemic is the continued dominance of the Omicron variant. Although the region saw a mixture of variants prior to the arrival of Omicron in November 2021, viral sequences have almost exclusively returned as Omicron and its subvariants ever since.

**Figure 3:**
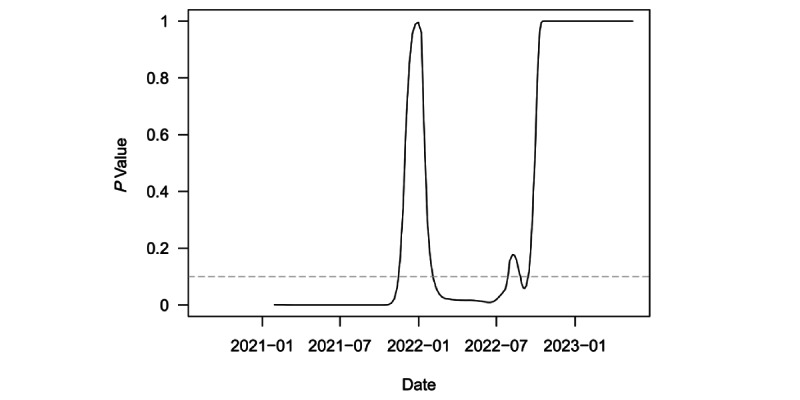
Rolling t-tests of weekly speed=10 over a 6-month window in LAC. LAC: Latin America and the Caribbean.

Figure 3 plots *P* values from a series of 1-sided *t* tests of whether the speed for the region was equal to or greater than the outbreak threshold of 10. These tests were conducted on a rolling 6-month window of the weekly regional speed. The dashed gray line denotes the least restrictive conventional significance level threshold of α=.10. The test immediately rejected the null hypothesis in favor of the alternative and continued to so until the end of 2021. The test statistic became totally insignificant for a brief period in December 2021, before regaining significance in February 2022. This second period of significance was driven by the large Omicron outbreak. The test lost significance again in August 2022 and has been totally insignificant from December 2022 onward. This more recent lack of statistical significance is consistent with the end to the pandemic in the region, as the test clearly failed to reject the null hypothesis of the outbreak threshold speed.

**Figure 4:**
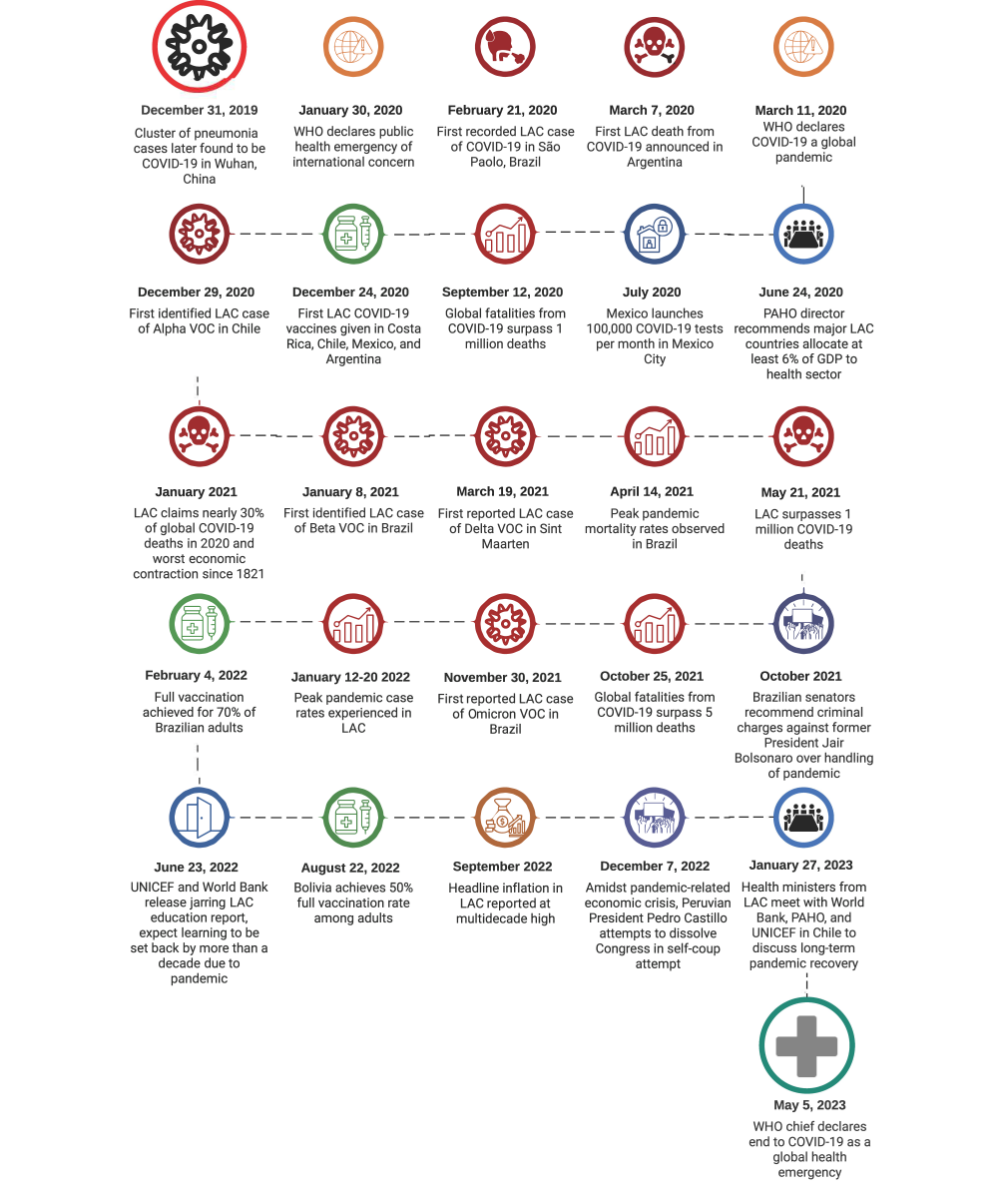
Timeline of the COVID-19 pandemic in LAC. LAC: Latin America and the Caribbean; PAHO: Pan-American Health Organization; VOC: variant of concern; WHO: World Health Organization.

Figure 4 provides a timeline of the onset of COVID-19 in LAC, as well as vaccination programs and major events that shaped the course of the pandemic in the region, such as economic, educational, and political impacts.

## Discussion

### Principal Findings

The results indicate that COVID-19 was well contained in LAC at the time of the WHO declaration. Both traditional and enhanced surveillance metrics indicated low and stable transmission rates around the time of the declaration. Nearly every SARS-CoV-2 test specimen returned as the Omicron variant from November 2021 onward. Finally, the rolling *t* test for regional outbreak status was entirely insignificant from December 2022 onward. Collectively, these results suggest a transition from pandemic to endemic in the region. However, these more recent trends do not reflect the full history of COVID-19 in LAC. The region had a unique set of risk factors and responses to COVID-19, which shaped the trajectory of transmission rates, especially in the early stages of the pandemic.

Although many countries in LAC reacted swiftly and preemptively to protect citizens and contain the spread of COVID-19, the region struggled to contain the levels of infection, mainly due to the prevalence of informal economies and the limitations of health infrastructure and social protection systems [86-90]. The informal sector of the economy refers to unrecorded economic activity, which would contribute to the gross domestic product (GDP) and tax revenue, if recorded. The sector contributes around a third of all economic activity in low-to-middle-income countries, but the sector poses a challenge for public health prevention efforts because its workers are often unknown and unregulated by governments [91]. LAC also faces an elevated risk profile for pandemics because of several factors, including climate change and human migration [92,93]. (We note, however, that the relationship between weather and COVID-19 transmissions can be complex, and not every product of climate change necessarily raises the risk of transmission [58]. Recent work indicates prevention strategies should be instituted in the summer and fully implemented in the winter [59].)

In May 2020, LAC was declared the epicenter of the pandemic by the WHO, with over 40% of the world’s COVID-19 deaths and more than 6.5 million confirmed cases at the time [63]. Just 1 year later, in May 2021, the region recorded its one-millionth COVID-19 death, with almost 90% of the deaths occurring in 5 countries: Brazil (44.3%), Mexico (22.1%), Colombia (8.3%), Argentina (7.3%), and Peru (6.7%) [94]. At that time, of the 153.5 million COVID-19 vaccines administered in the Americas, only 21.6% came through LAC [94,95]. Through 2022 and 2023, difficulties in production, access, and equitable distribution among different countries within LAC limited COVID-19 vaccination success and exacerbated hesitancy and rejection [96]. Despite the relatively late arrival of COVID-19 to LAC, the region suffered disproportionately. Several factors emerged as key determinants of the region’s hardship, including pandemic unpreparedness, fragile health care systems, socioeconomic inequalities, and poor governmental support [97,98]. Ultimately, LAC suffered the largest death toll from COVID-19 across developing regions and 1 of the sharpest declines in economic activity, with expected long-lasting socioeconomic consequences for the region [99].

LAC was economically vulnerable when the pandemic hit, due to limited fiscal resilience after the 2008 worldwide recession [86]. In 2020, the region experienced a historic 7% contraction in the GDP, with many countries seeing over a 10% economic decline [86,100]. This contraction was the largest in the region’s history [101]. Although modest growth was observed in 2021 and 2022 (6.9% and 3.5%, respectively), the International Monetary Fund projected a slowdown to 1.7% in 2023, largely due to inflation caused by interest rate hikes and fiscal policies [100]. Challenges included worsening inequality, higher poverty rates, and impacts on labor income, particularly among the extremely poor [102-106]. Major economic challenges anticipated for LAC in the years ahead include lingering effects of the pandemic in the setting of increased energy and food prices, which are mostly due to Russia’s invasion of Ukraine and China’s economic slowdown [100]. Even as the region rebounded, poverty rates remained high and continued to rise in 2022 [107,108]. Labor income, accounting for 68% of income among impoverished households, saw significant drops, particularly among the extremely poor [109].

LAC was especially burdened by sociopolitical unrest during the pandemic, in large part due to preexisting increases in authoritarian practices, weak democratic institutions, politicized judicial systems, and high levels of organized and unorganized crime and violence [100]. The region experienced a disproportionate number of political and public health threats, most notably the assassination of Haiti’s president in a coup d’état, followed by a devastating earthquake that effectively eliminated any remnants of the public health infrastructure [110,111]. COVID-19 was eclipsed by the outbreak of violence, cholera, and extreme poverty [112]. Other political turmoil included significant demonstrations in Brazil repeatedly protesting former President Jair Bolsonaro and his far-right administration’s handling of the pandemic, which included promotion of unscientific theories and pandemic denialism [113-116]. Intense protests also occurred in Cuba, Chile, Bolivia, and Colombia, with the average growth rate of the number of protests in LAC marked at 5.84% compared to a worldwide rate of 1.15%, according to the United Nations Development Program [97,117]. Given previously described socioeconomic issues, protests in LAC were also markedly intersectional, with examples including regular violent demonstrations in Colombia over both pandemic-related restrictions and police brutality and unpopular tax reform at the time [118]. Overall, in the region, as a result of unpopular policies and social unrest, a widespread anti-incumbent electoral trend was observed, starting in 2020 [100].

### Policies Implemented to Control and Mitigate the Transmission of COVID-19

Much of the LAC COVID-19 response was spearheaded by the WHO’s Pan American Health Organization (PAHO). PAHO efforts included building the COVID-19 Genomic Surveillance Regional Network, which provided the region a way to track the evolution of COVID-19 and monitor new variants and pathogens with pandemic potential [119]. PAHO also deployed extra COVID-19 support personnel and equipment to weaker regional health systems, notably in Venezuela, Bolivia, Paraguay, Guyana, Nicaragua, Honduras, Guatemala, Haiti, and some of the smaller Caribbean island states [120]. Throughout the pandemic, PAHO emphasized a multisectoral approach that included school closures, teleworking, hospital readiness assessments, and increased testing capabilities [120-124].

Specific countries’ public health responses to the early pandemic included Chile implementing a nightly curfew, with many large cities soon establishing mandatory quarantine measures [125]. After the first case was reported in Costa Rica on March 6, 2020, the country’s National Emergency Commission enabled systematic and interinstitutional mobilization of resources, as well as the activation of emergency operation centers [125]. After community transmission was identified on March 26, 2020, in Mexico, the country introduced mandatory restrictions that included suspending classes in all public and private schools, enforcing stay-at-home orders, cancelling public and private events, banning meetings of more than 100 people, and self-quarantining among disease contacts for at least 15 days [125]. Upon the declaration of a state of national emergency in Peru on March 15, 2020, strict controls were placed on citizens’ movements, with only trips related to the purchase of food and medicines allowed and only workers from critical sectors permitted to commute [125].

The WHO encouraged countries in LAC to deploy a phased vaccine introduction that prioritized frontline and health care workers, as well as older adults and people with underlying health conditions [126]. Vaccine introduction and administration, which began in the region at the end of December 2020, varied widely by country both initially and throughout the pandemic despite various organizational efforts to ensure equitable access [126,127]. Internal vaccine research and development in LAC was minimal compared to the rest of world, with no development spearheaded by any LAC nations [128]. The largest scientific contribution, as measured by published COVID-19 vaccine research manuscripts, was made by Brazil, which contributed one-third of the region’s papers regarding vaccine efficacy and safety [128]. Vaccination campaigns were a critical tool in the pandemic because vaccines were highly effective in reducing the probability of death, and population level fatality rates are partly a function of the percentage of the population vaccinated [62,129,130].

In October 2022, PAHO’s director reassured the region that disease trends insinuated a potential transition of the pandemic from an acute phase to a phase of sustained control [100]. Through 2022, the United States donated over 70 million COVID-19 vaccines to LAC directly and via the COVID-19 Vaccines Global Access (COVAX) partnership, with top recipients including Mexico (16.9 million doses), Guatemala (8.5 million doses), and Colombia (7 million doses) [100,131,132]. Larger agreements and agencies such as COVAX relied on a variety of vaccine types, mainly including messenger RNA (mRNA)–based vaccines (Pfizer-BioNTech, Moderna) and viral vector vaccines (Oxford University-AstraZeneca, Johnson & Johnson, Sputnik V) [133-135]. The lowest vaccine rates were observed in Haiti, with just over 2% of its population fully vaccinated by December 2022 [100]. Additional countries with vaccination rates below 40% included Grenada, Jamaica, St Lucia, and St Vincent and the Grenadines [100].

As the pandemic continued, new light was shed on the effect of LAC migrant crises on public health and vaccination policies [136]. Venezuela was deeply impacted [137,138]. By August 2022, more than 6.8 million Venezuelans fled the country due to political, social, and economic turmoil in what is now considered the largest international displacement in LAC in contemporary history [136]. Most Venezuelan refugees remained in LAC, with Argentina, Brazil, Chile, Colombia, Ecuador, and Peru having absorbed 75% of the migrants [139]. In addition to placing severe socioeconomic strain on recipient countries, the massive refugee influx also increased communicable disease spread and created new public health obstacles. For COVID-19, these challenges included ensuring access to vaccinations [136]. For example, most countries required government-issued identification to access COVID-19 vaccinations, which almost no refugees possessed upon arrival [136,140]. Colombia was 1 of few countries to eventually extend COVID-19 vaccine access to migrants with irregular immigration status, including those without documentation, and did so in October 2021 [136]. Meanwhile, many LAC leaders, including Colombian President Iván Duque Márquez and Venezuelan President Nicolás Maduro, blamed the constant flux of migrants throughout LAC for upsurges in COVID-19 cases [137]. In addition to questionable vaccine access, 75% of displaced Venezuelans in Peru and 39% in Colombia believed they would not be able to access health care if they became ill with COVID-19 [136].

At the end of the pandemic, to the best of our knowledge, barring Haiti due to the paucity of testing and reporting [141,142], the highest COVID-19 mortality rate in the region was in Peru, followed by Chile, Brazil, Trinidad and Tobago, and Argentina [100]. Despite accounting for just 25% of the world’s total COVID-19 case volume, LAC accounted for 43% of total deaths [119]. Major gaps in technical capacity, medical care coordination, and information technology infrastructure (many areas do not have infrastructure to support digital health systems and still rely on a pen-and-paper medical record system) were retrospectively highlighted as key reasons why LAC experienced such catastrophic spread and burden of COVID-19 [143,144]. In addition to these improvements, the region will focus on comprehensive disease surveillance strategies and methods to mitigate collateral damage on public health and social services during similar future crises [98].

### Limitations

COVID-19 data had become less frequently reported around the world by the time the WHO declared an end to the pandemic [145]. Additionally, more people began to use at-home tests as the pandemic evolved [146]. Some countries, such as Argentina, appear to have stopped reporting novel COVID-19 cases altogether. Viral specimen tests for VOCs in GISAID are also dependent on testing and sequencing capacity, which varied by country across the region. Because the enhanced surveillance metrics of the speed, acceleration, jerk, and 7-day persistence are based on rates, not total counts, statistical bias caused by countries dropping in or out of the sample is mitigated, but to the extent that a nonincluded country is unrepresentative of the region in the disease burden, the omission of a country or territory can still influence historical data comparisons. The dynamic panel estimates are based on a 120-day window, which further limits intertemporal biases caused by changes in reporting. Still, data availability is always a limitation. Continued reports on COVID-19 transmissions after mid-May 2023 would provide additional insight to whether COVID-19 had by then truly shifted from pandemic to endemic status. This team did not believe transmissions data were sufficiently available to draw firm conclusions beyond that time point.

### Conclusion

The concern about potential resurgences of the virus is valid. As long as COVID-19 continues to spread and mutate, the possibility of new variants emerging remains. Variants could potentially be more transmissible, resistant to vaccines, or cause more severe illness. This underlines the importance of continued vigilance, vaccination efforts, and worldwide cooperation to control the spread of the virus [54].

LAC experienced a high COVID-19 disease burden in the early stages of the pandemic. To prepare for future pandemics, one of the most important lessons from across the world may lie in the ways to reduce the disease burden ahead of the dissemination of vaccines and treatment modalities [147]. On that front, rapid and widespread testing of individuals, coupled with an epidemiological task force and contact-tracing system, is an effective first line of defense [148]. Lockdown policies have also proven effective [149], perhaps in part because international trade is positively associated with COVID-19 transmissions [61]. (As with climate change and transmissions, the relationship between containment policies and transmissions can be complex, and stricter containment may not always imply fewer transmissions [150,151].) Finally, new technologies will always renew the potential to improve early surveillance methods [152-154].

One bright point for LAC and other regions has been an advance in regional cooperation to tackle the public health threat posed by novel pathogens [155,156]. Novel indicators of pandemic preparedness at the regional level might further identify countries in relative need of support [157]. Measures of general governance, for example, are positively correlated with vaccination rates [60]. Recent work also suggests ways to reduce not only the death caused by novel pathogens but also the likelihood of their appearance, such as better surveillance of human and wildlife interaction and reductions in air pollution [158,159]. Continued cooperation will be necessary to reduce both the likelihood and impact of future pandemics [160,161].

## Appendix

Multimedia Appendix 1 Supplementary tables.

## Abbreviations

CDC: Centers for Disease Control and Prevention
COVAX: COVID-19 Vaccines Global Access
GDP: gross domestic product
GISAID: Global Initiative on Sharing All Influenza Data
LAC: Latin America and the Caribbean
mRNA: messenger RNA
PAHO: Pan American Health Organization
VOC: variant of concern
WHO: World Health Organization

## Data Availability

The data in this study were obtained from 2 publicly available sources. Our World in Data provided data on COVID-19 transmissions and death rates [162]. The Global Initiative on Sharing All Influenza Data (GISAID) provided data on sequenced SARS-CoV-2 specimens.
