# Supplementary material for: Updated Surveillance Metrics and History of the COVID-19 Pandemic (2020-2023) in Latin America and the Caribbean: Longitudinal Trend Analysis
Source: JMIR Public Health Surveill. 2024 May 17;10:e44398. doi: 10.2196/44398 (PMC11129782; doi:10.2196/44398)
Supplement: Multimedia Appendix 1 [file publichealth_v10i1e44398_app1.docx]

**Table S1. Static COVID-19 surveillance metrics for Latin American and Caribbean countries in the week of May 5, 2023.**

| Country | New COVID-19 cases, n | Cumulative COVID-19 cases, n | 7-day moving average of new cases | Weekly Transmission Rate per 100K individuals | New deaths, n | Cumulative deaths, n | 7-day moving average of deaths | Death rate per 100K individuals | Conditional death rate |
| --- | --- | --- | --- | --- | --- | --- | --- | --- | --- |
| Argentina | 0 | 10,044,957 | 0 | 0 | 0 | 130,472 | 0 | 0 | 0.01 |
| Aruba | 0 | 44,180 | 2.71 | 0 | 0 | 288 | 0.14 | 0 | 0.01 |
| Barbados | 0 | 107,566 | 14.29 | 0 | 0 | 589 | 0.14 | 0 | 0.01 |
| Belize | 0 | 70,782 | 0 | 0 | 0 | 688 | 0 | 0 | 0.01 |
| Bermuda | 0 | 18,860 | 0 | 0 | 0 | 165 | 0 | 0 | 0.01 |
| Bolivia | 49 | 1,197,650 | 54.86 | 2.82 | 0 | 22,380 | 0.43 | 0.02 | 0.02 |
| Brazil | 3,711 | 37,487,971 | 4,773.14 | 12.06 | 0 | 701,833 | 48.43 | 0.14 | 0.02 |
| Chile | 142 | 5,285,146 | 161.43 | 5.08 | 0 | 61,434 | 7.14 | 0.15 | 0.01 |
| Colombia | 100 | 6,365,262 | 93.29 | 1.35 | 0 | 142,722 | 1.29 | 0.01 | 0.02 |
| Costa Rica | 134 | 1,229,587 | 132.29 | 18.07 | 0 | 9,362 | 1.57 | 0.10 | 0.01 |
| Cuba | 32 | 1,113,400 | 39.86 | 0 | 0 | 8,530 | 0 | 0 | 0.01 |
| Curacao | 0 | 45,812 | 0.57 | 0 | 0 | 302 | 0.14 | 0 | 0.01 |
| Dominican Republic | 11 | 661,103 | 9.57 | 0 | 0 | 4,384 | 0 | 0 | 0.01 |
| Ecuador | 106 | 1,061,766 | 124.57 | 4.13 | 0 | 36,019 | 0 | 0 | 0.03 |
| Grenada | 0 | 19,693 | 0 | 0 | 0 | 238 | 0 | 0 | 0.01 |
| Guatemala | 184 | 1,248,823 | 129 | 0 | 0 | 20,196 | 1 | 0 | 0.02 |
| Guyana | 4 | 73,168 | 1.86 | 0 | 0 | 1,298 | 0 | 0 | 0.02 |
| Haiti | 0 | 34,228 | 0 | 0 | 0 | 860 | 0 | 0 | 0.03 |
| Honduras | 0 | 472,533 | 0 | 0 | 0 | 11,112 | 0 | 0 | 0.02 |
| Jamaica | 12 | 154,786 | 11.14 | 3.09 | 0 | 3,536 | 0 | 0.34 | 0.02 |
| Mexico | 1,036 | 7,598,737 | 1,083.71 | 5.69 | 0 | 334,036 | 6.57 | 0.03 | 0.04 |
| Nicaragua | 1 | 15,704 | 1 | 0.12 | 0 | 245 | 0 | 0 | 0.02 |
| Panama | 90 | 1,037,324 | 86.14 | 14.25 | 0 | 8,620 | 0 | 0.06 | 0.01 |

**Table S1 Continued.**

| Country | New COVID-19 cases, n | Cumulative COVID-19 cases, n | 7-day moving average of new cases | Weekly Transmission Rate per 100K individuals | New deaths, n | Cumulative deaths, n | 7-day moving average of deaths | Death rate per 100K individuals | Conditional death rate |
| --- | --- | --- | --- | --- | --- | --- | --- | --- | --- |
| Paraguay | 0 | 735,759 | 0 | 0 | 0 | 19,880 | 0 | 0 | 0.03 |
| Peru | 311 | 4,503,222 | 307.43 | 6.40 | 0 | 220,196 | 10.57 | 0.83 | 0.05 |
| Puerto Rico | 847 | 1,229,410 | 771.71 | 182.32 | 0 | 5,912 | 1.57 | 0.43 | 0 |
| St Barthelemy | 1 | 5,486 | 1 | 0 | 0 | 5 | 0 | 0 | 0 |
| Sint Maarten | 0 | 11,030 | 0 | 0 | 0 | 92 | 0 | 0 | 0.01 |
| St. Kitts & Nevis | 0 | 6,599 | 0.14 | 0 | 0 | 46 | 0 | 0 | 0.01 |
| St. Lucia | 0 | 30,052 | 0 | 0 | 0 | 409 | 0 | 0 | 0.01 |
| St. Vincent & Grenadines | 1 | 9,619 | 1 | 0 | 0 | 124 | 0 | 0 | 0.01 |
| Suriname | -8 | 82,495 | -9.57 | -8.99 | 0 | 1,404 | 0 | 0.85 | 0.02 |
| Trinidad & Tobago | 0 | 191,496 | 4.86 | 0 | 0 | 4,390 | 0.43 | 0 | 0.02 |
| Turks and Caicos Islands | 0 | 6,588 | 1 | 0 | 0 | 38 | 0 | 0 | 0.01 |
| United States Virgin Islands | 7 | 24,936 | 4.14 | 0 | 0 | 131 | 0.14 | 0 | 0.01 |
| Uruguay | 83 | 1,037,893 | 83.71 | 16.87 | 0 | 7,625 | 0 | 0.19 | 0.01 |
| Venezuela | 0 | 552,695 | 4 | 0 | 0 | 5,856 | 0 | 0 | 0.01 |

**Table S2. Novel COVID-19 surveillance metrics for Latin American and Caribbean countries for the week of May 5, 2023.**

| Country | Weekly Speed | Weekly Acceleration | Weekly Jerk | 7-day persistence effect on speed |
| --- | --- | --- | --- | --- |
| Argentina | 0 | 0 | 0 | 0 |
| Aruba | 8.86 | 0 | 0 | 0 |
| Barbados | 5.07 | 0 | 0 | 0 |
| Belize | 0 | 0 | 0 | 0 |
| Bermuda | 0 | 0 | 0 | 0 |
| Bolivia | 3.14 | -0.09 | -0.01 | 2.44 |
| Brazil | 15.52 | -0.98 | -0.13 | 13.62 |
| Chile | 5.76 | -0.34 | 0.08 | 6.86 |
| Colombia | 1.26 | 0.02 | 0.01 | 0.85 |
| Costa Rica | 17.88 | -0.11 | 0.12 | 14.38 |
| Cuba | 0.40 | 0 | 0 | 0 |
| Curacao | 2.05 | -0.96 | -0.04 | 4.15 |
| Dominican Republic | 0.07 | 0 | 0 | 0.03 |
| Ecuador | 4.84 | -0.11 | -0.09 | 2.95 |
| Grenada | 0 | 0 | 0 | 0 |
| Guatemala | 0.52 | 0 | 0 | 0.42 |
| Guyana | 0.11 | 0 | 0 | 0.36 |
| Haiti | 0 | 0 | 0 | 0 |
| Honduras | 0 | 0 | 0 | 0 |
| Jamaica | 2.79 | 0.12 | -0.01 | 1.31 |
| Mexico | 5.95 | -0.11 | 0.02 | 4.88 |
| Nicaragua | 0.11 | 0 | 0 | 0.06 |
| Panama | 13.68 | 0.08 | 0.07 | 9.94 |
| Paraguay | 0 | 0 | 0 | 0 |
| Peru | 6.32 | 0.09 | -0.04 | 3.80 |
| Puerto Rico | 166.10 | 5.43 | 0.01 | 91.89 |
| St Barthelemy | 0 | 0 | 0 | 0.91 |
| Sint Maarten | 0 | 0 | 0 | 0 |
| St. Kitts & Nevis | 0.30 | 0 | 0 | 0 |
| St. Lucia | 0 | 0 | 0 | 0 |
| St. Vincent & Grenadines | 1.10 | 0 | 0 | 0 |
| Suriname | 0 | 0 | 0 | 0 |
| Trinidad & Tobago | 2.21 | -0.43 | -0.32 | -0.53 |
| Turks and Caicos Islands | 7.19 | 0 | 0 | 0 |
| United States Virgin Islands | 2.44 | 0 | 0 | 1.21 |
| Uruguay | 17.11 | -0.14 | 0.04 | 13.33 |
| Venezuela | 0.06 | 0 | 0 | 0.01 |
| Argentina | 0 | 0 | 0 | 0 |
